# Effect of Mirror Therapy on Dexterity and Hand Grasp in Children Aged 9-14 Years with Hemiplegic Cerebral Palsy

**Published:** 2019

**Authors:** Akbar NARIMANI, Minoo KALANTARI, Hamid DALVAND, Seyyed Mahdi TABATABAEE

**Affiliations:** 1 Physiotherapy Research Center, Department of Occupational Therapy, school of Rehabilitation, Shahid Beheshti University of Medical Sciences, Tehran, Iran; 2 Department of Occupational Therapy, School of Rehabilitation, Shahid Beheshti University of Medical Sciences, Tehran, Iran; 3 Department of Occupational Therapy, School of Rehabilitation, Tehran University of Medical Sciences, Tehran, Iran; 4 Department of Basic Sciences, School of Rehabilitation, Shahid Beheshti University of Medical Sciences, Tehran, Iran

**Keywords:** Hemiplegic cerebral palsy, Mirror therapy, Upper limb function

## Abstract

**Objectives:**

Mirror therapy using visual feedback is one of the non-invasive methods along with other commonly used rehabilitation treatments for neurological patients which therapeutic effects on the affected upper limb of children with hemiplegic cerebral palsy have also been studied. We aimed to examine the effect of mirror therapy on improving the dexterity and grasp of children with hemiplegic cerebral palsy.

**Materials & Methods:**

In this single-blind clinical trial, 30 children with hemiplegic cerebral palsy in rehabilitation centers and special schools of Tabriz, northwest of Iran were randomly divided into two intervention and control groups in 2017. The children of the intervention group were under mirror therapy for 6 weeks. Occupational therapy exercise was done routinely for both groups. The grasp with dynamometer and the dexterity with box and block was measured. Data were analyzed using independent *t*-test and paired *t*-test.

**Results:**

The mean scores of the two groups in dexterity were significantly different after the intervention (*P*=0.008). However, there was no significant difference between the two groups in grasp.

**Conclusion:**

Mirror therapy in hemiplegic children is useful in improving the dexterity but not in improving of the grasp.

## Introduction

Pediatric cerebral palsy (CP) involves a group of progressive sensory and motor impairment as well as postural dysfunction caused by non-progressive damage to the immature brain (1). The prevalence of CP in the world is reported 2-3/1000 and in Iran, it is reported as 2.06/1000 live births (2, 3). One of the types of CP is spastic hemiplegia, known as half-body paralysis and the prevalence is about 35.1% of all children with CP (4). Upper limb function impairment is a common and disability consequence of problems in reaching, pointing, taking, releasing and manipulating objects (5). The ability to reach and grasp things is one of the basic tasks in activities of daily living (6). 

Among non-invasive approaches, mirror therapy is a relatively new approach that focuses on visual stimulation and the movement of limbs without damage. The reflection of the healthy limb movement in the mirror is thought the affected limb is moving naturally (7). In this method, the patient places his hands on the two sides of the mirror, so that the healthy hand is in front of the mirror and the affected hand behind the mirror. Thus, the patient understands the reflection of his healthy hand in the mirror as his affected hand (8). 

Mirror therapy, as a simple, inexpensive and, most importantly, a referral-based program, may improve the function of the limbs (9). This method does not require much activity and energy from the therapist, and the patient can even continue treatment at home after learning how to proceed (10).

Studies on the effect of mirror therapy on a variety of patients have shown that mirror therapy can improve the motor function of the upper extremity along with other rehabilitation programs. The effect of these studies on children in hemiplegia also shows that grasp and dexterity are significantly increased during a regular and continuous training program (11,12). Moreover, improving the range of motion and increasing the score of the Quest as well as Box and Block tests following is a mirror therapy (13, 14). In another study, mirror therapeutic exercises showed a significant difference in grasp and PDMS scale compared to the control group (15). 

In summary, there is little evidence of the effect of the mirror therapy in children with hemiplegic CP, and more studies have been done in a pilot or case study and are less than 10 yr old, therefore, we decided to investigate in clinical trial, the effect of mirror therapy on grasp and dexterity of the upper limb in children with hemiplegic CP aged 9-14 yr old.

## Materials & Methods

In this single-blind clinical trial, 30 children aged 9-14 yr old with a hemiplegic CP participated in rehabilitation centers and exceptional schools in Tabriz, northwest of Iran in 2017. The method of selecting subjects was simple randomized sampling. In this way, 30 children were selected and randomly divided into two groups: intervention and control group. The criteria for entry into this study included: diagnosis of hemiplegic CP by a pediatric neurologist, aged between 9 to14 yr, no cognitive impairment and unilateral neglect disorder, no orthopedic dysfunction and non-impairment verbal and visual acuity based on expert examination, no surgical procedures including tendon transfer, focusing attention on the mirror, and acquiring 1-3 of the MACS system and the tone of the affected upper limb less than or equal to 2, was corrected based on the modified Ashworth scale.

Our exclusion criteria were as follows: children who for any reason, do not have more than four alternate sessions or two consecutive sessions to participate in the program. Unwillingness of the child or his family to continue treatment at each stage of treatment. The child is unable to pay attention and attention during the treatment period. 

For both subjects, the routine occupational therapy program was used. In a routine occupational therapy program, several examples of new Bobath exercises, such as pressure tapping, inhibitory tapping, active, inactive and resistive flexion and extension of the elbow and wrists were used. In addition to the exercises, several functional exercises, such as cupping the mouth, pulling a circle, and scouring the towel were also carried out.

The intervention group received mirror therapy in addition to routine occupational therapy program, 3 d a week and 30 min daily for 6 wk. Children's referrals for occupational therapy were set up so that the two groups did not interact with each other during the study. For the mirror therapy, the child was placed in a sitting position on the chair and a 30*30 cm mirror was placed on the table in front of the child, with the affected hand behind the mirror, and the image of a healthy hand was easily seen in the mirror. Before the training began, the child was given the necessary training and guidance. The child focused on the image inside the mirror while practicing. The child was told to perform exercises bilateral and symmetrical as possible. These exercises included flexion and extension of the fingers and wrists, supination and pronation of forearm, and several functional exercises such as Lego removal, puzzle pieces, circle drawing, squeezing of special balls and towel cloth. Each move was performed 10 times and after about 20 sec of rest, the next move was taken.

 Assessments were made by an occupational therapist, which was not aware of the division of the groups. In order to evaluate the effectiveness of the mirror therapy on the upper limb of children before and after the end of treatment sessions, grasp and dexterity were evaluated. Box and Block tests were used to evaluate the dexterity. The reliability and validity of the test were shown (16), the number of blocks was recorded in one minute. In order to evaluate grasp, the Jamar Dynamometer was used. This measure has been studied for its reliability and validity (17), the child made a resistance grasp while the shoulder clinging to the trunk and the elbow at 90 degrees of flexion and forearm in the position of the neutrals and wrist in the 20-30 degree of extension. 

In this research, written and informed consent was obtained from each participating child. The present study was approved by the Ethics Committee of Shahid Beheshti University of Medical Sciences and its ethical code is IR.SBMU.RETECH.REC.1396.409 and IRCT code is IRCT20180627040262N1.

The Kolmogorov-Smirnov test was used to determine the normal status of the data. Regarding the fact that all variables had normal distribution in both control and intervention groups, *t*-test and paired *t*-test were used for comparison between mean and standard deviation before and after intervention.

## Results

Thirty children with hemiplegic CP participated in this study (Table 1). There was no significant difference between the mean age of two groups (*P*=0.80). Sexual distribution of the two groups was homogeneous (*P*=0.71).

**Table 1:** The demographic and clinical features of hemiplegic cerebral palsy in both group

	Group		
		Mirror therapy (n=15)	Control (n=15)
Age (yr) (mean + SD)		10.84 + 1.62	11.30 + 1.49
Sex (%)	Female Male	7 (46.7) 8 (53.3)	6 (40) 9 (60)
Affected limb	Right Left	10 (66.7) 5 (33.3)	7 (46.7) 8 (53.3)
Modified Ashworth (%)	1	6 (40)	5 (33.3)
	1^+^	6 (40)	8 (53.3)
	2	3 (20)	2 (13.4)
MACS (%)	I	6 (40)	4 (26.7)
	II	5 (33.3)	8 (53.3)
	III	4 (26.7)	3 (20)

The mean and standard deviation of dexterity were calculated in both groups before and after the intervention. There was no significant difference between the two groups in the dexterity before intervention, but there was a significant difference after the mirror therapy (Table 2).

**Table 2:** Pre and Post Dexterity measure in the treatment and control groups

Groups	Pre-Test	Post-Test	t	p
Therapy group, mean ± SD	24.73±4.81	28.46±4.53	- 5.28	< 0.001
Control group, mean ± SD	22.60±5.30	23.40±5.22	- 1.92	0.07
t	-1.15	- 2.83		
p	0.25	0.008		

The mean and standard deviation of grasp were calculated in both groups before and after the intervention. There was no significant difference between the two groups in the grasp before and after intervention (Table 3). 

**Table 3:** Pre and Post Grasp Measure in the treatment and control groups

Groups	Pre-Test	Post-Test	t	p
Therapy group, mean ± SD	3.46 ± 1.35	4.44 ± 1.60	- 7.55	< 0.001
Control group, mean ± SD	3.68 ± 1.41	3.86 ± 1.53	- 2.50	0.02
t	0.42	- 1.01		
p	0.67	0.32		

## Discussion

The aim of this study was to determine the effect of mirror therapy on the grasp and dexterity of children with hemiplegic CP compared to control group. Mirror therapy had a much better effect than routine exercises on the dexterity function of children with CP. 

Dexterity scores were significantly increased, and the results of this study come in agreement with these results (14). The results of studying the grasp of children with CP in two groups of intervention and control showed that there was no significant difference between the two groups in grasp improvement. The results of this study are not consistent with the study on increasing the grasp of children (18). A similar study was performed on 90 hemiplegic children who after 5 wk the use of mirror treatment did not show any significant difference between the control and intervention groups in dexterity improvement (19). However, in a clinical trial study, the dexterity scores obtained in the control and intervention groups showed significant (20). 

The results of this study indicate positive advantages of the combination of mirror therapy with conventional rehabilitation techniques in order to maximize patient's function improvement. Since the mirror therapy technique is a therapeutic technique based on the functioning of the central nervous system, improvement in this study could be due to the activation of the inactive nerves after injury. Several fundamental mechanisms accounted for the effect of mirror therapy on their motor skills have been identified. The mirror illusion created from the natural movement of the affected hand may be an alternative to reduce the proprioceptive information (21). For patients, looking at the mirror offers visual data, which leads to the reconstruction and rehabilitation of their premotor cerebral cortex by the connection formed between the visual input areas and premotor. 

The mirror therapy may also help the patient to use the affected hand in daily activities by reversing the non- use learning process (20). Visual illusions can provoke the primary motor cortex (7). In addition, bilateral arm training has been suggested as a possible mechanism for the efficacy of the mirror therapy. when the nonparetic limb engaged during motor training, crossed facilitatory drive from the intact hemisphere increase excitability in motor pathways of the paretic limb (11).

The effect of mirror therapy on some of the indicators of motor abilities has not been supported by some studies, which led to the contradictory results. The bimanual movements of the mirrors result in a certain negative impact on the bimanual performance of cerebral palsy (22). Mirror therapy can improve the upper extremity motor function of the affected limb through increased activity of motor neurons and reduce the movement disorder to its minimum, which is in line with the results of our study (23-24). 

In the present study, all children were given counseling and training to do better exercises. Following these tutorials and recommendations in the process of improving children is very effective, but the degree of adherence of children to these teachings and recommendations may vary, cited by the limitations of this research. Another limitation of this study was the attention and concentration of patients to the non-affective limb image in the mirror. Although patients did not have cognitive impairment, some patients had a high concentration, while others were unable to concentrate their maximum sensation on the non-affective limb image in the mirror, which could also affect the results of the study this method is protected in neurophysiology and can be used as a simple, inexpensive and usable method at home. In order to generalize the results of this study, more clinical trials and sample size are needed.

**In conclusion, **the method of mirror therapy is the techniques of rehabilitation to maximize patient's healing. Since the mirror therapy is based on the function of the central nervous system, the improvement in this study could be due to the activation of the inactive nerves after injury, although this should be considered in the following studies. The mirror therapy is performed as a simple, inexpensive and affordable treatment at home mirror therapy is an effective additional tool to the rehabilitation program for children with hemiparesis, to improve hand dexterity. Future researchers are recommended to investigate the effect of mentioned exercise on other motor variables such as eye-hand coordination.
